# Altered cochlear innervation in developing and mature naked and Damaraland mole rats

**DOI:** 10.1002/cne.24682

**Published:** 2019-03-25

**Authors:** Catherine M. Barone, Sytse Douma, Daniël O. J. Reijntjes, Brigitte M. Browe, Christine Köppl, Georg Klump, Thomas J. Park, Sonja J. Pyott

**Affiliations:** ^1^ Laboratory of Integrative Neuroscience, Department of Biological Sciences University of Illinois at Chicago Chicago Illinois; ^2^ Department of Otorhinolaryngology and Head/Neck Surgery University of Groningen, University Medical Center Groningen Groningen The Netherlands; ^3^ Cluster of Excellence “Hearing4All”, Department of Neuroscience, School of Medicine and Health Sciences Carl von Ossietzky University Oldenburg Oldenburg Germany; ^4^ Research Center Neurosensory Science Carl von Ossietzky University Oldenburg Oldenburg Germany

**Keywords:** auditory, BK channels, cochlea, development, gerbil, inner and outer hair cells, inner ear, lateral and medial efferent olivocochlear innervation, mice, presynaptic afferent ribbons, RRID: AB_11150471, RRID: AB_143157, RRID: AB_2200400, RRID: AB_2313725, RRID: AB_2534069, RRID: AB_2535810, RRID: AB_399431, RRID: AB_90710, tonotopy

## Abstract

Compared to many other rodent species, naked mole rats (*Heterocephalus glaber*) have elevated auditory thresholds, poor frequency selectivity, and limited ability to localize sound. Because the cochlea is responsible for encoding and relaying auditory signals to the brain, we used immunofluorescence and quantitative image analysis to examine cochlear innervation in mature and developing naked mole rats compared to mice (*Mus musculus*), gerbils (*Meriones unguiculatus*), and Damaraland mole rats (*Fukomys damarensis*), another subterranean rodent. In comparison to mice and gerbils, we observed alterations in afferent and efferent innervation as well as their patterns of developmental refinement in naked and Damaraland mole rats. These alterations were, however, not always shared similarly between naked and Damaraland mole rats. Most conspicuously, in both naked and Damaraland mole rats, inner hair cell (IHC) afferent ribbon density was reduced, whereas outer hair cell afferent ribbon density was increased. Naked and Damaraland mole rats also showed reduced lateral and medial efferent terminal density. Developmentally, naked mole rats showed reduced and prolonged postnatal reorganization of afferent and efferent innervation. Damaraland mole rats showed no evidence of postnatal reorganization. Differences in cochlear innervation specifically between the two subterranean rodents and more broadly among rodents provides insight into the cochlear mechanisms that enhance frequency sensitivity and sound localization, maturation of the auditory system, and the evolutionary adaptations occurring in response to subterranean environments.

## 1 INTRODUCTION

Naked mole rats (*Heterocephalus glaber*) are long‐lived rodents that live in large eusocial colonies in narrow, underground burrows. Their subterranean environment restricts oxygen levels, light exposure, and acoustic stimulation and has led to various physiological adaptations (Hetling et al., 2005; Park et al., 2017). Some of these adaptations might involve neotenous retention of immature characteristics by prolonged and/or arrested development (Larson & Park, 2009; Skulachev et al., 2017). The unique life history of naked mole rats has made them of enormous interest to comparative biologists and biomedical researchers. Nonetheless, their auditory system has been relatively uninvestigated. Further investigation of the structure and function of the auditory system in this species would provide comparative insight into the anatomical and physiological mechanisms that shape auditory responses throughout the lifespan of an organism.

Compared to many other rodent species, naked mole rats have relatively high auditory thresholds, poor high frequency hearing, and a limited ability to localize sound. For comparison, mice (*Mus musculus*) hear frequencies extending from 1 kHz to about 100 kHz, with the most sensitive frequency in the audiogram being approximately 16 kHz (Heffner & Heffner, 1993). Gerbils (*Meriones unguiculatus*) hear frequencies ranging from 125 Hz to about 60 kHz, with the most sensitive frequency being approximately 2 kHz (Müller, 1996; Ryan, 1976). Both mice and gerbils localize sound effectively, with mice using primarily interaural level differences and pinna cues (Allen & Ison, 2010; Heffner, Koay, & Heffner, 2001) and gerbils taking additional advantage of interaural time differences (Tolnai, Beutelmann, & Klump, 2017). In contrast, naked mole rats hear within a more restricted range of frequencies, from 125 Hz to 8 kHz, with only slightly more sensitive hearing at 4 kHz (Heffner & Heffner, 1993; Okanoya et al., 2018), and, moreover, have very limited ability to localize sound (Heffner & Heffner, 1993). Although hearing in Damaraland mole rats, another subterranean mole rat, has not been investigated, other *Fukomys* species show reduced cochlear tuning (Kössl, Frank, Burda, & Muller, 1996), elevated auditory thresholds and frequency ranges restricted to between 125 Hz and 4 kHz (five octaves), with best hearing within 0.8 and 1.4 kHz (Gerhardt, Henning, Begall, & Malkemper, 2017).

Previous work in naked mole rats has documented anatomical differences in their peripheral and central auditory structures, but how these differences contribute to comparative differences in their auditory function is unclear. Most conspicuously, naked and Damaraland mole rats lack pinnae, external structures of the outer ear that funnel sound and provide localization cues. Structural differences in the middle and inner ear of naked mole rats were also recently characterized by Mason, Cornwall, and Smith (2016) using microcomputed tomography and dissections. The authors of this study concluded that these structural differences were not by themselves sufficient to explain poor high frequency hearing in naked mole rats. Moreover, naked mole rats did not show specializations (most notably, hypertrophy of the malleus) that support low frequency hearing by inertial bone conduction (i.e., seismic hearing). Centrally, naked mole rats possess all major auditory brainstem nuclei (Gessele, Garcia‐Pino, Omerbasic, Park, & Koch, 2016; Heffner & Heffner, 1993) except the superior paraolivary nucleus (Gessele et al., 2016). However, the structures involved in binaural processing of sound and sound localization are comparatively smaller (Gessele et al., 2016; Heffner & Heffner, 1993). The molecular organization of these structures has been investigated only more recently and only centrally. Perineuronal nets, specialized extracellular matrix structures responsible for synaptic stabilization in the adult brain, are observed in the auditory brainstem nuclei in naked mole rats and show no differences compared to other rodents (Beebe & Schofield, 2018). Nonetheless, molecular differences were reported in another study: specifically, the entire superior olivary complex lacked membrane‐bound expression of the hyperpolarization‐activated channel HCN1 (Gessele et al., 2016), an ion channel that greatly contributes to binaural temporal precision in the superior olivary complex of other rodents (Khurana et al., 2012; Koch, Braun, Kapfer, & Grothe, 2004).

In this study, we were particularly interested in examining the anatomical and molecular organization of cochlear innervation in the auditory sensory epithelium (organ of Corti) of naked mole rats because this innervation is responsible for encoding and relaying auditory signals to the brain and, moreover, shapes the development of auditory brainstem structures (O'Neil, Connelly, Limb, & Ryugo, 2011). We hypothesized that differences in the innervation of the organ of Corti, perhaps resulting from reduced postnatal maturation, contribute to differences in auditory function in naked mole rats in comparison to other rodents. To begin to test this hypothesis, we used immunofluorescence to examine patterns of afferent and efferent innervation in excised organs of Corti from mature and developing naked mole rats compared to mice, gerbils, and also Damaraland mole rats (*Fukomys damarensis*). This selection of species allowed comparison of naked mole rats to rodents with sensitive high frequency hearing (mouse), sensitive low frequency hearing (gerbils) and another more closely related, subterranean, eusocial mole rate (Damaraland mole rats). We observed alterations in afferent and efferent innervation as well as their patterns of developmental refinement in naked mole rats compared to both mice and gerbils and also compared to Damaraland mole rats. Differences in cochlear innervation specifically between the two subterranean rodents and more broadly among rodents provides insight into the evolutionary adaptations to subterranean environments as well as the cochlear mechanisms that contribute to hearing sensitivity and sound localization.

## 2 MATERIALS AND METHODS

### 2.1 Animals

Animal protocols conformed to the respective legislation at the University of Illinois at Chicago, the University Medical Center Groningen (the Netherlands), the University of Groningen (the Netherlands), and the University of Oldenburg (Germany) outlined specifically in the *Guide for the Care and Use of Laboratory Animals*, the *Policy on Humane Care and Use of Laboratory Animals*, and Article 54 of Directive 2010/63/EU. Naked mole rats (*H. glaber*) and Damaraland mole rats (*F. damarensis)* were obtained from colonies maintained at the University of Illinois at Chicago. Mice (C57BL6 and FVB/NJ; *M. musculus*) were obtained from colonies maintained at the University Medical Center Groningen. Gerbils (*M. unguiculatus*) were obtained from colonies maintained at the University of Oldenburg. A total of 29 mice, 7 gerbils, 18 naked mole rats, and 4 Damaraland mole rats were used. Ages of animals are indicated in Table 1. For mice and gerbils exact ages were known and given in the text. For naked and Damaraland mole rats exact ages are given when known. Adult Damaraland mole rats were approximately 5 years of age and referred to as “mature” in the text.

**Table 1:** Number of afferent ribbons per HC at given ages in mice, gerbils, naked mole rats, and Damaraland mole rats^a^

Species	Age	Ribbons/IHC	Ribbons/OHC
Mouse (C57BL6)	P6	41.5 ± 1.7 (*N* = 4)	22.2 ± 0.9 (*N* = 2)
	P9	33.7 ± 0.7 (*N* = 3)	18.7 ± 0.8 (*N* = 2)
	P12	19.5 ± 0.6 (*N* = 4)	2.5 ± 0.03 (*N* = 3)
	P15	16.4 ± 1.1 (*N* = 4)	2.3 ± 0.08 (*N* = 3)
	P21	17.7 ± 0.5 (*N* = 3)	2.3 ± 0.08 (*N* = 3)
	P42 (6 weeks)	17.0 ± 0.7 (*N* = 7)	2.3 ± 0.04 (*N* = 4)
Gerbil	P9	41.0 ± 3.5 (*N* = 3)	16.8 ± 0.9 (*N* = 3)
	P42 (6 weeks)	25.2 ± 2.2 (*N* = 3)	2.8 ± 0.08 (*N* = 4)
Naked mole rat	P7	19.3 ± 3.7 (*N* = 3)	24.4 ± 0.09 (*N* = 3)
	P11	19.2 ± 1.8 (*N* = 3)	17.6 ± 0.2 (*N* = 3)
	P14	19.6 ± 1.8 (*N* = 3)	13.2 ± 0.8 (*N* = 3)
	P28	15.5 ± 1.8 (*N* = 2)	9.7 ± 0.8 (*N* = 2)
	P182 (6 months)	12.9 ± 0.5 (*N* = 3)	6.9 ± 0.2 (*N* = 3)
	P365 (1 year)	13.6 ± 1.2 (*N* = 4)	7.1 ± 0.7 (*N* = 4)
Damaraland mole rat	P5	16.26 (*N* = 1)	12.0 (*N* = 1)
	Mature	13.9 ± 1.0 (*N* = 3)	14.5 ± 0.9 (*N* = 3)

Abbreviations: HC = hair cell; IHC = inner hair cell; OHC = outer hair cell.

a *N* = total number of individuals.

For comparison, the onset of hearing in mice and gerbils occurs around 2 weeks of age but auditory maturation continues until weaning (Ehret, 1976; Finck, Schneck, & Hartman, 1972). Mice wean at 3–4 weeks of age and begin reproductive activity between 6 and 8 weeks of age (Prichett & Taft, 2007). Gerbils also wean between 3 and 5 weeks of age and reach reproductive activity at 4 months of age (Elwood, 1975). Although the exact age at the onset of hearing is not known in either naked or Damaraland mole rats, naked mole rats do hear by 1 year of age (Heffner & Heffner, 1993). Naked and Damaraland mole rats wean at 4 weeks of age (Jarvis, O'Riain, Bennett, & Sherman, 1994) and reach reproductive activity between 12 and 18 months of age (Edrey, Hanes, Pinto, Mele, & Buffenstein, 2011; Jarvis et al., 1994).

To obtain cochleae, animals were deeply anesthetized by either isoflurane inhalation (University of Groningen and University of Illinois at Chicago) or injection of a lethal dose of pentobarbital (University of Oldenburg) and then rapidly decapitated. Procedures published previously were used to isolate intact organs of Corti from cochleae for immunofluorescence (Braude et al., 2015; McLean, Smith, Glowatzki, & Pyott, 2009). To prevent differences in antigen detection due to differences in processing, identical fixation and immunostaining conditions were used for all specimens. Specifically, whole cochleae were isolated from the temporal bone in ice cold phosphate‐buffered saline (PBS) and immediately placed into ice cold 4% paraformaldehyde (PFA) diluted in PBS. Cochleae were fixed in 4% PFA/PBS for exactly 1 hr at 4 °C. Organs of Corti were then dissected from the cochleae in ice cold PBS and treated with a blocking buffer (PBS with 5% normal goat serum, 4% Triton X‐100, and 1% saponin) for 1 hr at room temperature. Turns were incubated in the primary antibody diluted in blocking buffer overnight at room temperature and then rinsed three times for 10 min in PBS with 0.2% Triton X‐100 (PBT). After rinsing, the turns were then incubated in the secondary antibody diluted in blocking buffer for 4 hr at room temperature. Samples were then rinsed three times for 10 min in PBT, one time in PBS, and mounted on glass slides in Vectashield mounting medium (Vector Labs). All incubations and rinses were performed on a rocking table.

### 2.2 Antibody characterization, immunofluorescence, and confocal microscopy

Immunofluorescent staining was performed using mouse monoclonal (IgG1) anti‐CTBP2 (RRID: AB_399431; aa 361–445 from mouse CTBP2; 612044, BD Bioscience; 1:300), rabbit polyclonal antibody against the GluR2/3 (RRID: AB_90710; EGYNVYGIESVKI from rat GluR2; AB1506; Merck Millipore; 1:300), rabbit polyclonal anti‐synapsin I (RRID: AB_2200400; Synapsin Ia/b from bovine brain; AB1543; Merck Millipore; 1:500), mouse monoclonal (IgG2A) anti‐synapsin Ia/b (RRID: AB_11150471; aa 2–29 from human Synapsin Ia/b N‐terminus; sc‐376622; Santa Cruz Biotechnology; 1:300), and rabbit polyclonal anti‐BK channel (RRID: AB_2313725; aa 1097–1196 from mouse KCa1.1; APC‐021; Alomone Labs; 1:500). The specificity of the primary antibodies was verified by the vendors. Secondary antibodies, AlexaFluor 488 goat anti‐mouse IgG1 (RRID: AB_2534069), AlexaFluor 568 goat anti‐rabbit (RRID: AB_143157), and AlexaFluor 647 goat anti‐mouse IgG2A (RRID: AB_2535810), were purchased from Invitrogen and used diluted 1:500.

Low magnification micrographs of entire organs of Corti were obtained with a Leica DM 4000B fluorescence microscope. High magnification confocal micrographs of identified tonotopic regions of the organ of Corti were acquired using a Leica SP8 confocal microscope with a 60× Olympus PlanApo oil‐immersion lens (NA 1.42) under the control of the Leica Application Suite software. Image stacks were collected to encompass the entire region of interest from the specified frequencies. The step size (optical section thickness) was approximately 0.3 μm and determined by stepping at half the distance of the theoretical *z*‐axis resolution (the Nyquist sampling frequency). Images were acquired in a 1,024 × 1,024 pixel raster at a sampling speed of 100 Hz. The laser power, PMT voltage, and gain and offset adjustments were adjusted to optimize the dynamic range of the detected intensity values. Images are presented as *z*‐projections through the collected optical stacks.

Unless specifically stated, data are collected from the 16 kHz region in mice and from the 2 kHz region in gerbils. These regions represent the frequencies of best hearing and were identified using previously determined cochlear place‐frequency maps (Müller, 1996; Müller, von Hunerbein, Hoidis, & Smolders, 2005). For both mice and gerbils, these regions are approximately 50% along the length of the basilar membrane. The total basilar membrane (cochlear canal) length is approximately 6.8 mm in mice and 12.1 mm in gerbils (Kirk & Gosselin‐Ildari, 2009). Because similar cochlear place‐frequency maps have not been determined for either naked or Damaraland mole rats, we compared to equivalent positions (50%) along the length of the basilar membrane. The total basilar membrane length is approximately 5.8 mm in naked mole rats (Mason et al., 2016) and 10.9 mm in *Fukomys micklemi* (Mason et al., 2016) and 11.1 mm in *Fukomys anselli* (Kirk & Gosselin‐Ildari, 2009), two relatives of the Damaraland mole rat.

### 2.3 Image analysis

Three‐dimensional (3D) reconstructions were obtained using Imaris 6.4 software (Bitplane Inc.) and used to determine the number, volume, and location of synaptic elements. To determine the number of synaptic elements per hair cell, the Imaris spots function was used to detect immunopuncta within a given field of view. This value was then divided by the total number of hair cells within that field of view. Hair cell counts were obtained from counts of immunofluorescently detected hair cell nuclei (for CTBP2 immunolabeling). For organs of Corti from naked and Damaraland mole rats, CTBP2 immunolabeling was not restricted to the nuclei of the hair cells but also apparent in the nuclei of other cell types. In 3D reconstructions, the hair cell nuclei were identified by their generally larger size and proximity to the CTBP2‐immunolabeled afferent ribbons. In addition, differential interference contrast images obtained in parallel aided identification of the hair cells. Volumes (μm^3^) of immunopuncta were determined using the Imaris surfaces function. All numerical values and their 3D locations were exported from Imaris for further statistical analyses.

Custom script developed in *R* was used to calculate Euclidean distances between immunopuncta (as described previously in Sadeghi, Pyott, Yu, & Glowatzki, 2014; Ye, Goutman, Pyott, & Glowatzki, 2017) and to classify inner hair cell (IHC) afferent ribbons (CTBP2 immunopuncta) as either pillar or modiolar. Briefly, for this later analysis, *x*, *y*, and *z* coordinates were determined for the afferent ribbons and the nuclei using the spots function in Imaris. Using principal component analysis, (eigen) vectors were fit to the coordinates defining the row of afferent ribbons and also the coordinates defining the nuclei. The minimum and maximum *x*, *y*, *z* coordinates for the vector fitting the afferent ribbons and the mean *x*, *y*, *z* coordinate for the vector fitting the coordinates of the nuclei determined the plane that defined the pillar‐modiolar axis. The pillar and modiolar sides of this plane were determined from other landmark structures (e.g., the spiral ganglion neurons and outer hair cells [OHCs]). The *x*, *y*, *z* coordinates of the individual afferent ribbons were then used to classify ribbons as belonging to either the pillar or modiolar side. To allow comparison across *z*‐stacks, ribbon volumes, determined using the Imaris surfaces function, from a given stack were normalized to the median volume from that stack.

### 2.4 Statistics

Statistical analyses were performed using SPSS 24. Data for independent samples were analyzed using a general linear model ANOVA. In case of repeated measurements from the same individual subject (e.g., when obtaining data from multiple hair cells in a subject) a mixed model ANOVA was applied taking the subject into account. Post hoc pairwise tests used the Bonferroni correction, which was not applied in planned comparisons. Differences were considered significant when *P* < 0.05 (*) and highly significant when *P* < 0.01 (**). Group results are reported as the mean ± *SEM*. In most cases, *n* represents the number of individuals.

## 3 RESULTS

### 3.1 Afferent innervation in the organs of Corti from mature mice, gerbils, naked, and Damaraland mole rats

**Figure 1:**
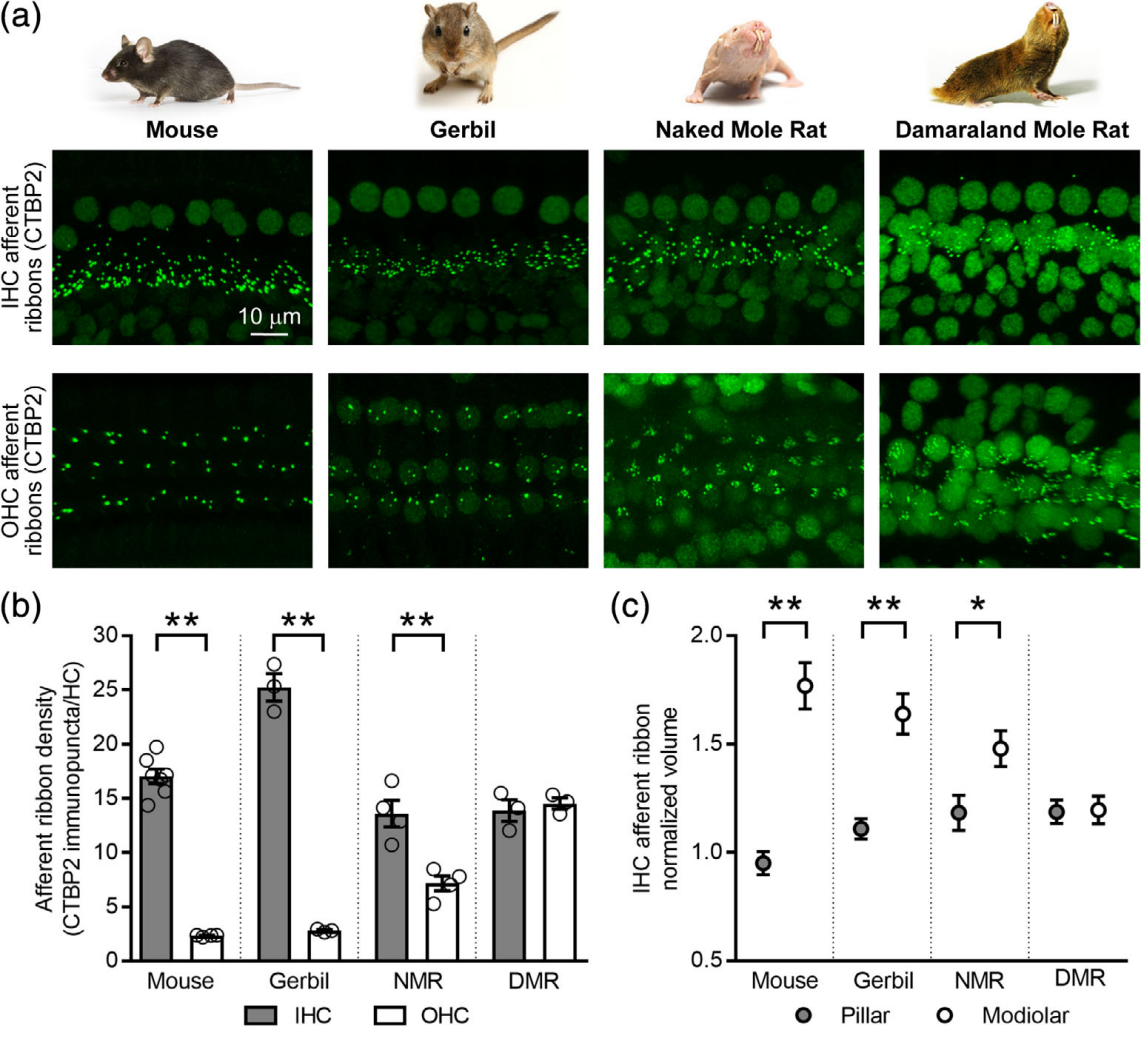
Comparison of inner and outer hair cell afferent innervation in mature mice (6 weeks old), gerbils (6 weeks old), naked mole rats (1 year old), and Damaraland mole rats (mature). (a) Projections through *z*‐stacks of confocal sections spanning the regions of the organ of Corti containing the inner hair cell (IHC) and outer hair cell (OHC) afferent ribbons immunolabeled with an antibody against CTBP2 (green). (b) Comparison of afferent ribbon density CTBP2 immunpuncta per IHC (gray bars) and OHC (white bars). (c) Comparison of the mean normalized volume of pillar (gray circles) and modiolar (white circles) afferent ribbons. Asterisks indicate significantly different values (*P < 0.05, **P < 0.01). Values are provided in Tables 1 and 2

In the mammalian organ of Corti, there are two classes of sensory hair cells (Goutman, Elgoyhen, & Gomez‐Casati, 2015). The IHCs are responsible for relaying information to the brain for the perception of sound and make afferent connections to the type I spiral ganglion neurons. The OHCs are responsible for generating the cochlear amplifier and make afferent connections to the type II spiral ganglion neurons. We quantified the numbers of afferent synapses in these two types of sensory hair cells in the mature organs of Corti from mice (C57BL6, 6 weeks old) gerbils (6 weeks old), naked mole rats (1 year old), and Damaraland mole rats (mature) by immunolabeling with an antibody against CTBP2, a protein enriched in the presynaptic afferent ribbons. In all animals, both classes of hair cells show CTBP2‐immunoreactivity indicative of presynaptic afferent ribbons (green, Figure 1a).

To compare afferent ribbon numbers per hair cell both within and across these four species, we quantified the number of afferent ribbons (CTBP2 immunopunta) per IHC (gray bars, Figure 1b) and per OHC (white bars, Figure 1b). Quantification shows that, when comparing within species, the number of afferent ribbons per IHC compared to OHC was significantly greater in mice, gerbils, and naked mole rats but not Damaraland mole rats. In addition, there were differences in the numbers of afferent ribbons per IHC and OHC across species. Gerbils had significantly greater numbers of ribbons per IHC compared to all other species. Damaraland mole rats had significantly greater numbers of afferent ribbons per OHC compared to all other species. Naked mole rats also had significantly greater numbers of afferent ribbons per OHC compared to both mice and gerbils. Values are provided in Table 1. Although not quantified, there were no apparent systematic differences in the numbers of afferent ribbons per OHC row in any of the four species investigated.

We also investigated postsynaptic afferent glutamate receptors by immunolabeling with a rabbit polyclonal antibody against GluR2/3. In mice and gerbils, GluR2/3 immunoreactivity is punctate and juxtaposed to presynaptic afferent ribbons. In contrast to mice and gerbils, we observed no GluR2/3 immunoreactivity in naked or Damaraland mole rats (data not shown). Postsynaptic glutamate receptor labeling was not characterized further.

We were also interested in the distribution of the IHC afferent ribbons and ribbon sizes along the pillar‐modiolar axis of the IHCs (Figure 1c). In cat, pillar‐modiolar differences in IHC afferent ribbon sizes correlate with pillar‐modiolar differences in afferent fiber thresholds (Merchan‐Perez & Liberman, 1996). Pillar‐modiolar differences in IHC afferent ribbon sizes have also been documented in mice (Liberman, Wang, & Liberman, 2011) and gerbils (Zhang, Engler, Koepcke, Steenken, & Koppl, 2018). To compare differences between the numbers and volumes of pillar and modiolar IHC afferent ribbons, we localized and quantified the volumes of ribbons (CTBP2 immunopunta) as described in Section 2. In mice, there were fewer pillar ribbons (44%) than modiolar ribbons (56%). In contrast, in gerbils, naked mole rats, and Damaraland mole rats, the fractions of pillar and modiolar ribbons were equivalent. When comparing the mean normalized volumes of the pillar (gray circles, Figure 1c) versus modiolar (white circles, Figure 1c) IHC afferent ribbons, values within species were significantly different in mice, gerbils, and naked mole rats but not Damaraland mole rats. The steepness of this gradient (i.e., the ratio between the mean volumes of the pillar versus modiolar afferent ribbons) also appeared to vary across species, with the steepest gradients observed in mice. Values are provided in Table 2.

**Table 2:** Normalized volume of pillar and modiolar afferent ribbons in mature mice, gerbils, naked mole rats, and Damaraland mole rats^a^

Species	Age	Pillar afferent ribbons	Modiolar afferent ribbons
Mouse (C57BL6)	P42 (6 weeks; *N* = 4 individuals)	0.95 ± 0.05 (*N* = 232)	1.77 ± 0.11 (*N* = 293)
Gerbil	P42 (6 weeks; *N* = 3 individuals)	1.12 ± 0.08 (*N* = 270)	1.64 ± 0.09 (*N* = 262)
Naked mole rat	P182 (6 months; *N* = 3 individuals)	1.18 ± 0.08 (*N* = 142)	1.48 ± 0.08 (*N* = 143)
Damaraland mole rat	Mature (*N* = 3 individuals)	1.19 ± 0.05 (*N* = 172)	1.20 ± 0.06 (*N* = 176)

a *N* = total number of individuals and afferent ribbons examined.

To summarize, Damaraland mole rats are particularly unique among the rodent species examined: unlike mice, gerbils, and naked mole rats, Damaraland mole rats show the same number of afferent ribbons per hair cell in both IHCs and OHCs and lack the pillar‐modiolar gradient in IHC afferent ribbons sizes observed in other rodents.

### 3.2 Tonotopic variations in afferent ribbon density and BK channel expression in the IHCs from mature mice, gerbils, and naked mole rats

In both mice and gerbils, IHC afferent ribbon density peaks where the cochlea is most sensitive to sound (i.e., the most sensitive frequency in the audiogram; Meyer et al., 2009). Thus, reduced tonotopic variation in IHC afferent ribbon density could contribute to the poor sensitivity of hearing across frequencies observed in naked mole rats (Heffner & Heffner, 1993). Therefore, we analyzed IHC afferent ribbon density along the length of the cochlear spiral. In parallel, we investigated tonotopic changes in BK channel expression. BK channels are large conductance, voltage‐, and calcium‐activated potassium channels that are present in both inner and OHCs in the mammalian cochlea (reviewed in Pyott & Duncan, 2016). BK channel expression in both types of hair cells shows increasing expression from apical (low frequency) to basal (high frequency) regions following the onset of hearing in a variety of animals investigated (Pyott & Duncan, 2016). Therefore, the presence of BK channels in mammalian hair cells is associated with both frequency tuning of the cochlea and functional maturation of the cochlea. For these reasons, we were interested in whether BK channels were also present in IHCs from naked more rats, and, if so, if BK channel expression together with afferent ribbon density varied tonotopically.

**Figure 2:**
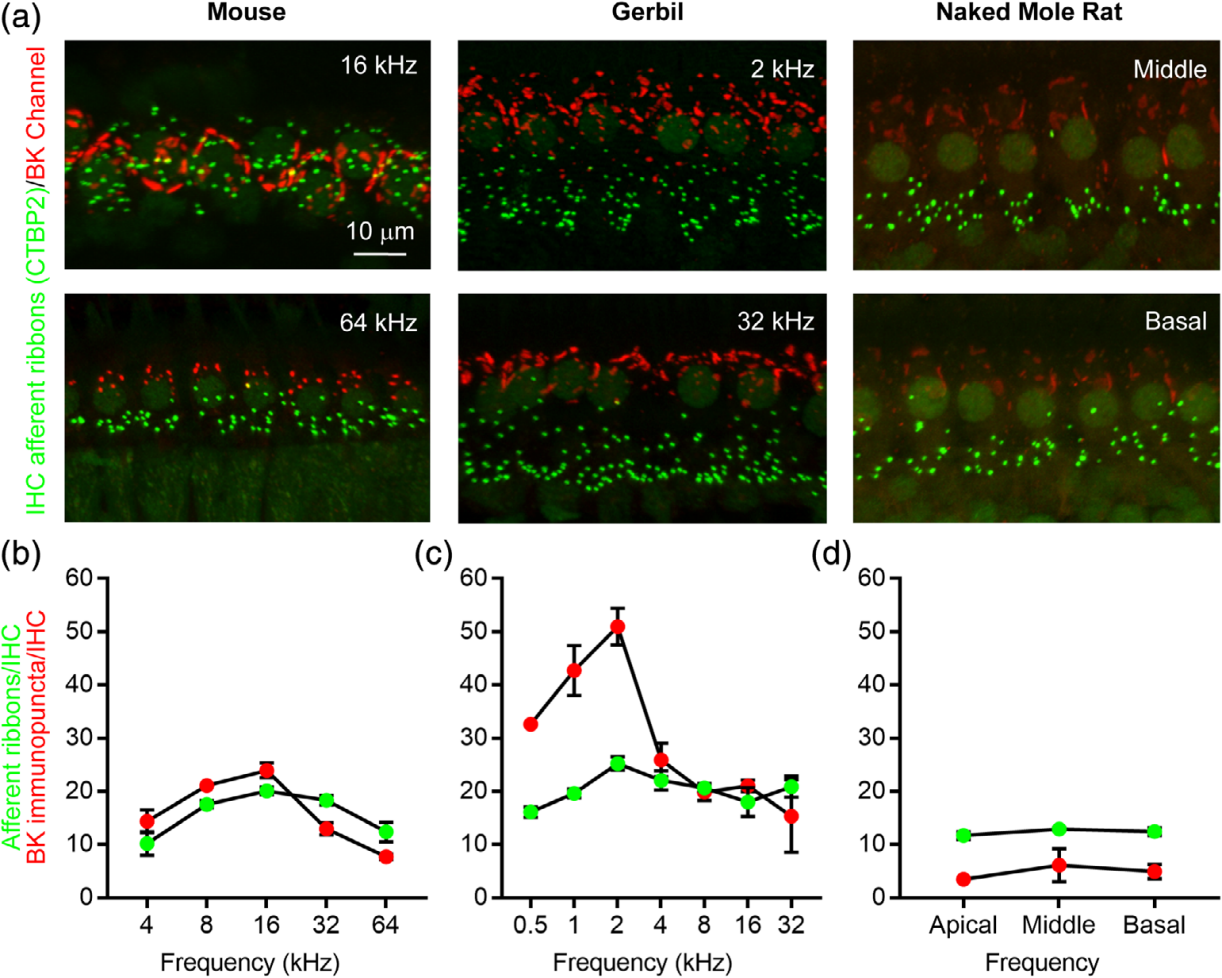
Tonotopic comparison of afferent ribbon density and BK channel expression in inner hair cells from mature mice, gerbils, and naked mole rats. (a) Projections through *z*‐stacks of confocal sections spanning the regions of the organ of Corti containing the inner hair cell (IHC) afferent ribbons immunolabeled with an antibody against CTBP2 (green) and BK channels immunolabled with an antibody against the C‐terminus (red). Mice and gerbils are 6 weeks old, naked mole rats are 6 months old, and Damaraland mole rats are mature. Tonotopic regions are indicated. (b–d) Mean afferent ribbon density (CTBP2 immunopuncta/IHC, green) and BK channel density (BK immunopuncta/IHC, red) are plotted as a function of the tonotopic position in mice (b), gerbils (c), and naked mole rats (d). Values are provided in Table 3

To examine afferent ribbon density and BK channel expression tonotopically in the IHCs, we immunolabeled organs of Corti isolated from mice (6 weeks old), gerbils (6 weeks old), and naked mole rats (6 months) with a mouse monoclonal antibody against CTBP2 (green, Figure 2a) and a rabbit polyclonal antibody against the BK channel (red, Figure 2a). These experiments utilized wild‐type littermates of BK channel knockout mice, which are on the FVB/NJ background and have been previously used to examine BK channel expression in the IHCs (Pyott et al., 2007; Pyott, Glowatzki, Trimmer, & Aldrich, 2004; Rohmann, Wersinger, Braude, Pyott, & Fuchs, 2015; Wersinger, McLean, Fuchs, & Pyott, 2010). For all tonotopic regions examined in these animals, BK channel immunoreactivity was punctate and clustered around the neck of the hair cells and away from the synaptic pole where afferent ribbons were localized. (This separation is not always apparent in the 2D projections through the 3D *z*‐stacks shown in Figure 2a.)

To examine expression quantitatively, we determined the number of afferent ribbons and BK channel immunopuncta per IHC at defined tonotopic locations in mice and gerbils and in apical, middle, and basal turns in naked mole rats (Figure 2b). These values are provided in Table 3. Although the sample size was not sufficient for statistical comparisons, a few trends were visible from the data. First, in mice and gerbils, the number of afferent ribbons and BK channel immunopuncta per IHC increased steadily with frequency and then dropped again at frequencies beyond the most sensitive frequency (16 kHz in mice and 2 kHz in gerbils). Second, in both mice and gerbils, the number of BK channel immunopuncta per IHC was greater than the number of afferent ribbons per IHC for frequencies at and below the most sensitive frequency before becoming smaller than the number of afferent ribbons per IHC for frequencies above the most sensitive frequency. Third, in naked mole rats, the number of afferent ribbons and BK channel immunopuncta per IHC showed little tonotopic variation from apical to middle and then middle to basal turns, and, furthermore, the number of BK channel immunopuncta per IHC was smaller than the number of afferent ribbons per IHC for all three turns. Thus, in contrast to mice and gerbils, naked mole rats show no evidence of tonotopic variation in either IHC afferent ribbon density or BK channel expression.

**Table 3:** Tonotopic variation in BK channel expression and afferent ribbons in IHCs from mice, gerbil, and naked mole rats^a^

Species (age)	Frequency (kHz or turn)	Ribbons/IHC	BK channel immunopuncta/IHC
Mouse (FVB/NJ, 6 weeks)	4	10.2 ± 2.2 (*N* = 4)	14.4 ± 2.2 (*N* = 4)
	8	17.6 ± 0.6 (*N* = 3)	21.1 ± 0.8 (*N* = 3)
	16	20.1 ± 0.7 (*N* = 4)	24.0 ± 1.4 (*N* = 4)
	32	18.3 ± 0.9 (*N* = 4)	13.0 ± 1.1 (*N* = 4)
	64	12.4 ± 1.8 (*N* = 4)	7.7 ± 0.5 (*N* = 4)
Gerbil (6 weeks)	0.5	16.1 ± 1.0 (*N* = 4)	32.6 ± 0.3 (*N* = 4)
	1	19.6 ± 0.8 (*N* = 4)	42.8 ± 4.7 (*N* = 4)
	2	25.2 ± 2.2 (*N* = 3)	51.0 ± 3.5 (*N* = 3)
	4	22.1 ± 1.8 (*N* = 3)	25.9 ± 3.1 (*N* = 3)
	8	17.4 ± 3.2 (*N* = 3)	19.9 ± 1.6 (*N* = 3)
	16	18.0 ± 2.7 (*N* = 3)	14.7 ± 4.0 (*N* = 3)
	32	20.9 ± 2.0 (*N* = 3)	15.4 ± 6.8 (*N* = 3)
Naked mole rat (6 months)	Apical	12.5 ± 0.7 (*N* = 3)	3.5 ± 0.6 (*N* = 3)
	Middle	12.9 ± 0.5 (*N* = 3)	6.1 ± 5.4 (*N* = 3)
	Basal	11.7 ± 0.7 (*N* = 3)	5.0 ± 1.4 (*N* = 2^b^)

Abbreviations: IHC = inner hair cell; OHC = outer hair cell.

a *N* = total number of individuals examined.

b One replicate showed very faint BK channel labeling that was not quantified.

### 3.3 Lateral and medial efferent innervation in the organs of Corti from mature mice, gerbils, naked, and Damaraland mole rats

**Figure 3:**
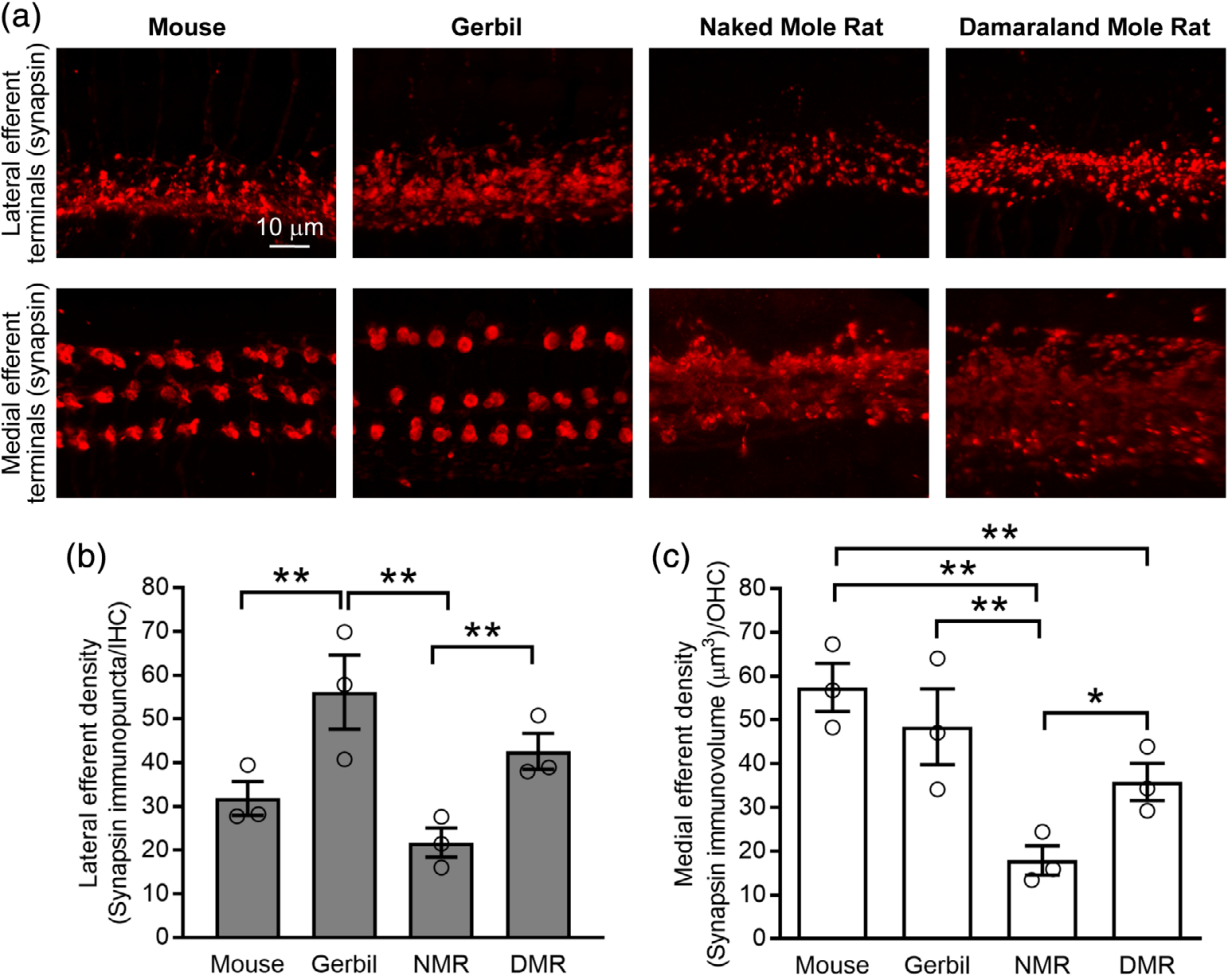
Comparison of lateral and medial efferent innervation in mature mice, gerbils, naked mole rats, and Damaraland mole rats. (a) Projections through *z*‐stacks of confocal sections spanning the regions of the organ of Corti containing the lateral and medial efferent terminals immunolabeled with an antibody against synapsin (red). Mice and gerbils are 6 weeks old, naked mole rats are 1 year old, and Damaraland mole rats are mature. (b) Comparison of lateral efferent density, synapsin immunopuncta per inner hair cell (IHC). (c) Comparison of medial efferent density, synapsin immunovolume per outer hair cell (OHC). Asterisks indicate significantly different values (*P < 0.05, **P < 0.01). Values are provided in Table 4

Efferent innervation of the hair cells begins during development and persists, with significant maturational modifications, in the adult cochlea. In the mature mammalian cochlea, there are two olivocochlear efferent systems (Guinan, 2006). The medial olivocochlear component projects to the OHCs and serves to regulate OHC activity and the cochlear amplifier. The lateral olivocochlear component contacts the afferent dendrites contacting the IHCs, but its role is less understood. We investigated efferent innervation of the IHCs and OHCs by immunolabeling organs of Corti isolated from mature mice (6 weeks old), gerbils (6 weeks old), naked mole rats (1 year old), and Damaraland mole rats (mature) with a rabbit polyclonal antibody against synapsin (red, Figure 3a). Lateral efferent innervation (in the region below the IHCs) is present, abundant, and organized into relatively small terminals in all four species (Figure 3a upper panels). Medial efferent innervation (in the region of the OHCs) is also present in all four species (Figure 3a lower panels). In mice and gerbils, efferent terminals are relatively large and organized into discrete rows defined by the OHCs. In contrast, medial efferent terminals are noticeably smaller and less organized in naked and Damaraland mole rats.

To compare lateral efferent innervation density across the four species, we quantified the number of efferent terminals (synapsin immunopunta) per IHC (Figure 3b). Because medial efferent terminals differed substantially in size between mice and gerbils compared to naked and Damaraland mole rats, we quantified the volume of efferent terminals (synapsin immunopunta) per OHC to compare medial efferent innervation density across the four species (Figure 3c). Lateral efferent innervation density varied both between individuals and across species but was significantly reduced in naked mole rats compared to both gerbils and Damaraland mole rats. Moreover, lateral efferent innervation density was significantly reduced in mice compared to gerbils. Medial efferent innervation density also varied both between individuals and across species. Medial efferent innervation density was significantly reduced in naked mole rats compared to all other species, and medial efferent innervation density was significantly reduced in Damaraland mole rats compared to mice. Values are provided in Table 4. When comparing the number of medial efferent terminals per OHC across species, we observed an average of 1.2 (255/205), 2.3 (480/204), 3.7 (718/192), and 5.4 (923/171) terminals/OHC in gerbils, mice, naked, and Damaraland mole rats, respectively. These findings indicate that medial efferent terminals are smaller but more numerous in naked and Damaraland mole rats compared to mice and gerbils.

**Table 4:** Efferent innervation density in mature mice, gerbils, naked mole rats, and Damaraland mole rats^a^

Species	Age	Lateral efferent terminals/IHC	Medial efferent terminal volumes/OHC
Mouse (C57BL6)	6 weeks (*N* = 3)	31.8 ± 3.8	57.4 ± 5.5
Gerbil	6 weeks (*N* = 3)	56.1 ± 8.4	48.4 ± 8.7
Naked mole rat	1 year (*N* = 3)	21.7 ± 3.3	17.9 ± 3.4
Damaraland mole rat	Mature (*N* = 3)	42.6 ± 4.1	35.8 ± 4.3

Abbreviations: IHC = inner hair cell; OHC = outer hair cell.

a *N* = total number of individuals examined.

### 3.4 Developmental changes in postnatal afferent innervation in the organs of Corti from mice, gerbils, naked, and Damaraland mole rats

Mammals undergo stereotyped patterns of postnatal maturation of cochlear innervation shortly before the onset of hearing (Yu & Goodrich, 2014). In mice and gerbils, postnatal maturation of afferent synapses involves pruning of supernumerary afferent ribbons between the spiral ganglion neurons and the IHCs and OHCs shortly before the onset of hearing (Yu & Goodrich, 2014). Because previous experiments showed prolonged patterns of synapse refinement in hippocampal and olfactory structures of the brain in naked mole rats beyond 1 year of age (Penz et al., 2015) and because our experiments showed reduced BK channel expression in IHCs from naked mole rats (Figure 2), we also wanted to test whether delayed postnatal maturation of cochlear innervation might underlie alterations in cochlear innervation observed in mature naked and Damaraland mole rats.

**Figure 4:**
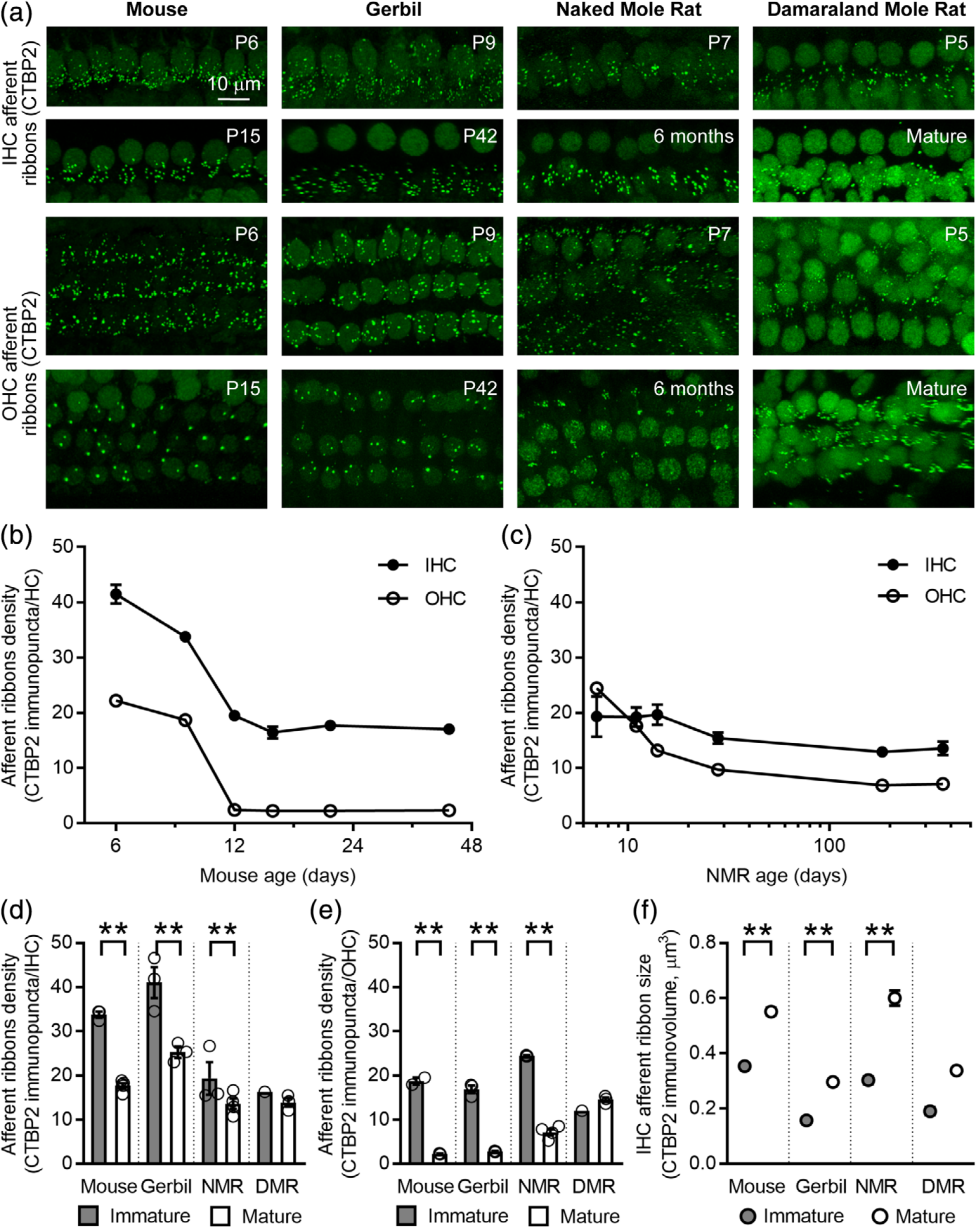
Comparison of afferent development in mice, gerbils, naked mole rats, and Damaraland mole rats. (a) Projections through *z*‐stacks of confocal sections spanning the regions of the organ of Corti containing the inner hair cell (IHC) and outer hair cell (OHC) afferent ribbons immunolabeled with an antibody against CTBP2 (green). Developmental ages are indicated. (b) Afferent ribbon density, CTBP2 immunpuncta per hair cell (HC), during development in mice. Onset of hearing in mice occurs at the end of the second postnatal week. (c) Afferent ribbon density, CTBP2 immunpuncta per HC, during development in naked mole rats. Ages are scaled logarithmically. The onset of hearing in naked mole rats is unknown. (d) Comparison of IHC afferent ribbon density in immature (gray bars) and mature (white bars) mice, gerbils, naked mole rats, and Damaraland mole rats. (e) Comparison of OHC afferent ribbon density in immature (gray bars) and mature (white bars) mice, gerbils, naked mole rats, and Damaraland mole rats. (f) Comparison of the mean volume of IHC afferent ribbon volumes in immature (gray circles) and mature (white circles) mice, gerbils, naked mole rats, and Damaraland mole rats. Asterisks indicate significantly different values (**P < 0.01). Values are provided in Tables 1 and 5

To examine the postnatal elimination of afferent synapses, we immunolabeled the afferent ribbons in organs of Corti isolated from variously aged mice, gerbils, naked mole rats, and Damaraland mole rats with a mouse monoclonal antibody against CTBP2 (green, Figure 4a). Both the IHCs and OHCs from mice and gerbils showed conspicuously more afferent ribbons at younger ages before the onset of hearing compared to older ages after the onset of hearing. In contrast, the difference in numbers of IHC and OHC afferent ribbons between younger ages, during the first postnatal week, and older ages, 6 months in naked mole rats and mature Damaraland mole rats, was much less conspicuous than in mice and gerbils.

To examine the time course of postnatal afferent ribbon pruning, we quantified the number of afferent ribbons (CTBP2 immunopunta) per hair cell at various ages in both mice (Figure 4b) and naked mole rats (Figure 4c). In mice, between the first and third postnatal week (P6 and P21), afferent ribbons per IHC were reduced approximately two‐fold and afferent ribbons per OHC were reduced more dramatically, approximately ten‐fold. In mice, the numbers of afferent ribbons per IHC and afferent ribbons per OHC were significantly greater at P6 compared to P9 and the numbers at both P6 and P9 were significantly greater compared to P12, P21, and P42. These data indicate that the pruning of afferent ribbons per IHC and OHC is complete by the end of the second postnatal week (P12), and the numbers of afferent ribbons per hair cell remain stable thereafter. In naked mole rats, between the first postnatal week and the first year of life (P7 to P365), afferent ribbons per IHC and OHC showed less reduction compared to mice. No significant differences were observed comparing afferent ribbons per IHC across all ages but the small sample size may have reduced the statistical power. When comparing afferent ribbons per OHC, numbers were significantly reduced from P7 to P11, P11 to P14, and P14 to P28. After P28, no significant differences were observed. These data indicate that the pruning of afferent ribbons per OHC is complete by the end of the fourth postnatal week (P28), and the numbers of afferent ribbons per OHC remain stable thereafter. Values are provided in Table 1.

For comparison across all four species, we additionally quantified the number of afferent ribbons (CTBP2 immunopunta) per hair cell for immature and mature gerbils (P9 and P42/6 weeks) and Damaraland mole rats (P5 and mature) compared to immature and mature mice (P7 and P21) and immature and mature naked mole rats (P7 and 1 year). When comparing within species for these two ages (and, hence, without Bonferroni correction for multiple comparisons as in Figure 4c), mice, gerbils, and naked mole rats showed significant reductions in the number of afferent ribbons per IHC (Figure 4d). Mice, gerbils, and naked mole rats also showed significant reductions in the number of afferent ribbons per OHC (Figure 4e). In contrast, no evidence of developmental pruning of either IHC or OHC afferent ribbons was observed in Damaraland mole rats (Figure 4d,e). However, because only one P5 Damaraland mole rat sample was available, no statistical comparisons were made between immature and mature Damaraland mole rats. Values are provided in Table 1.

Finally, in addition to quantifying the pruning of hair cell afferent ribbons, we also examined developmental changes in the size of these afferent ribbons. In mice, the afferent ribbons show a developmental increase in size that accompanies their overall reduction in number (Sobkowicz, Rose, Scott, & Slapnick, 1982; Wong et al., 2014). When comparing within species, IHC afferent ribbons from mice, gerbils, and naked mole rats were significantly larger at mature ages (Figure 4f). Damaraland mole rats also showed a developmental increase in IHC afferent ribbon volumes with maturation. However, because only one P5 Damaraland mole rat sample was available, no statistical comparisons were made between immature and mature Damaraland mole rats. Values are provided in Table 5. The normalization of ribbon volume used throughout our study precludes any comparison across different ages. Therefore, absolute ribbon volumes were used only for this specific developmental question.

**Table 5:** Volume of inner hair cell ribbons at given developmental ages in mice, gerbils, naked mole rats, and Damaraland mole rats^a^

Species	Age	Volume (μm^3^)
Mouse (C57BL6)	P9	0.354 ± 0.006 (*N* = 2,270, 3 individuals)
	P21	0.551 ± 0.014 (*N* = 1,187, 3 individuals)
Gerbil	P9	0.158 ± 0.005 (*N* = 1,920, 3 individuals)
	P42 (6 weeks)	0.297 ± 0.009 (*N* = 1,301, 3 individuals)
Naked mole rat	P7	0.303 ± 0.014 (*N* = 886, 3 individuals)
	P182 (6 months)	0.601 ± 0.027 (*N* = 450, 3 individuals)
Damaraland mole rat	P5	0.190 ± 0.015 (*N* = 274, 1 individual)
	Mature	0.338 ± 0.011 (*N* = 363, 3 individuals)

a *N* = total number of afferent ribbons and individuals examined.

### 3.5 Developmental changes in postnatal efferent innervation in the organs of Corti from mice, gerbils, naked, and Damaraland mole rats

**Figure 5:**
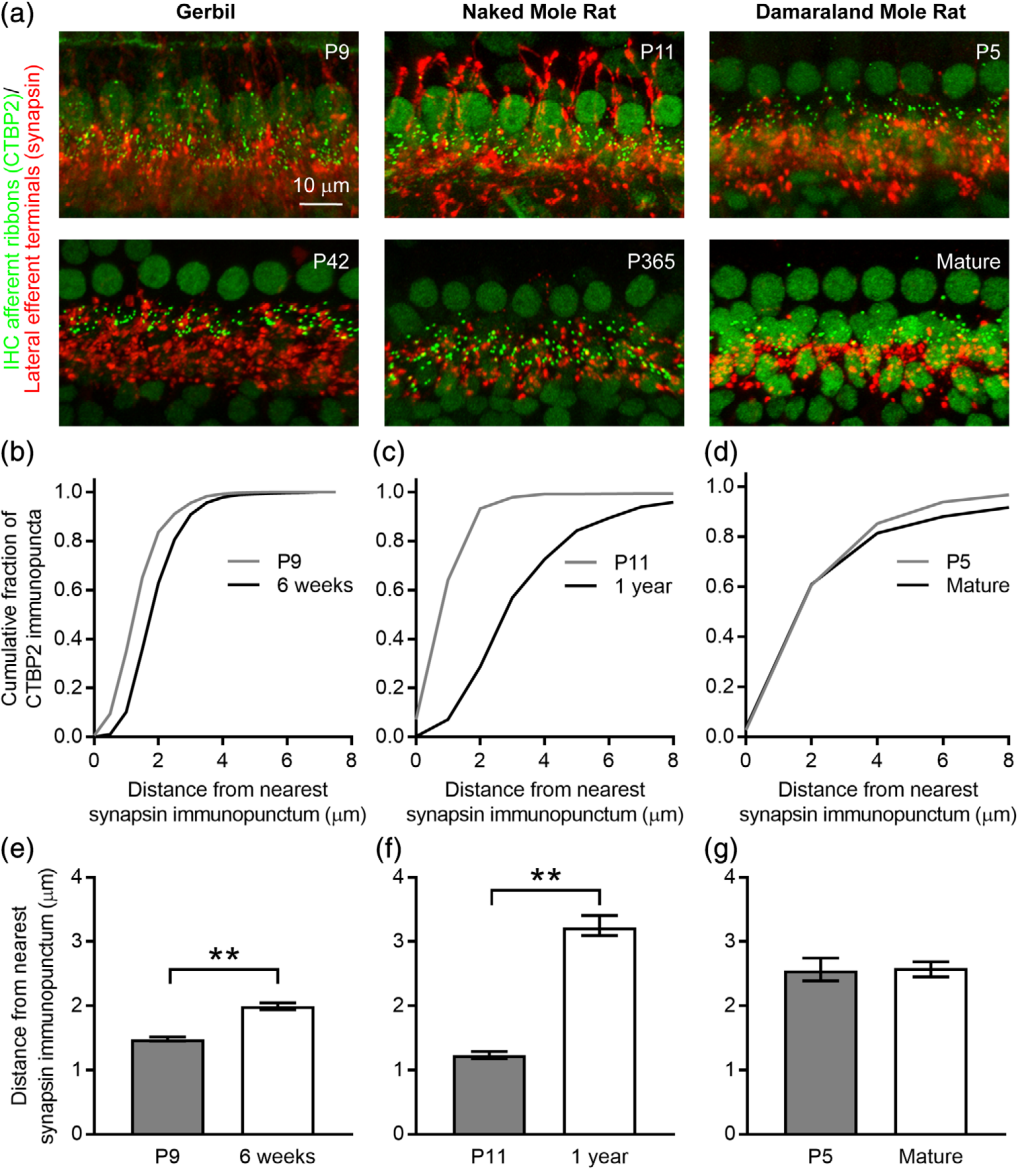
Comparison of efferent development in gerbils, naked mole rats, and Damaraland mole rats. (a) Projections through *z*‐stacks of confocal sections spanning the regions of the organ of Corti containing the inner hair cell (IHC) and outer hair cell (OHC) afferent ribbons immunolabeled with an antibody against CTBP2 (green) and efferent terminals immunolabled with an antibody against synapsin (red). Developmental ages are indicated. Note that medial efferent terminals on OHC are not included within the *z*‐range shown. (b–d) Cumulative histograms plotting the Euclidean distance between each CTBP2 immunopunctum and its nearest synapsin immunopunctum in immature (gray lines) and mature (black lines) gerbils, naked mole rats, and Damaraland mole rats. (e–g) Mean Euclidean distance between each CTBP2 immunopunctum and its nearest synapsin immunopunctum in immature (gray bars) and mature (white bars) gerbils, naked mole rats, and Damaraland mole rats. Asterisks indicate significantly different values (**P < 0.01). Values are provided in Table 6

Observations in mice and gerbils (as well as rats and hamsters) indicate that efferent innervation of the mammalian cochlea undergoes extensive modification shortly before the onset of hearing, generally around the second postnatal week (Simmons, 2002). As part of this maturation, efferent terminals retract from the IHCs as medial efferent contacts onto the IHCs are lost and lateral efferent contacts onto the IHC afferent terminals consolidate. To assess postnatal maturation of efferent innervation in the naked mole rat and Damaraland mole rat, we immunolabeled organs of Corti isolated from P9 and P42 (6 week old) gerbils, P11 and 1 year old naked mole rats, and P5 and mature Damaraland mole rats with a rabbit polyclonal antibody against synapsin to label presynaptic efferent terminals (red, Figure 5a) and a mouse monoclonal antibody against CTBP2 to label IHC afferent ribbons and nuclei (green, Figure 5a). In both gerbils and naked mole rats, but less obviously in Damaraland mole rats, efferent terminals underwent extensive developmental reorganization. In young gerbils and naked mole rats, efferent terminals could be seen in the regions both above and below the IHC afferent ribbons and nuclei, consistent with efferent terminals contacting the IHCs. In contrast, in older gerbils and naked mole rats, efferent terminals were largely consolidated to the region below the IHC afferent ribbons and nuclei at the base of the IHCs. (This separation is not always apparent in the 2D projections through the 3D *z*‐stacks shown in Figure 5a.) In contrast, in Damaraland mole rats, efferent terminals were largely consolidated to the region below the IHC afferent ribbons and nuclei at the base of the IHC even at the younger age.

To quantify the degree of developmental retraction, the distance between each afferent ribbon (CTBP2 immunopunctum) and its nearest efferent terminal (synapsin immunopunctum) was quantified as described previously (Sadeghi et al., 2014; Ye et al., 2017). We predicted that this distance would increase with development. Cumulative distributions plotting the Euclidean distance between each IHC afferent ribbon and its nearest efferent terminal at the younger (gray line) and older (black line) ages showed a rightward shift with development for both gerbils (Figure 5b) and naked mole rats (Figure 5c). This rightward shift indicates an increase in the distance between nearest neighbor afferent ribbon and efferent terminal pairs, consistent with the loss of medial efferent contacts onto IHCs and consolidation of lateral efferent contacts onto the IHC afferent terminals below the IHCs. In contrast, this increase in the distance between nearest neighbor afferent ribbon and efferent terminal pairs was not observed in Damaraland mole rats (Figure 5d). When comparing within species, in gerbils and naked mole rats, the mean Euclidean distances were significantly different between the two ages examined (Figure 5e–f). In contrast, the mean Euclidean distances were not obviously different between the two ages in Damaraland mole rats (Figure 5g). However, because only one P5 Damaraland mole rat sample was available, no statistical comparisons were made between immature and mature Damaraland mole rats. Values are provided in Table 6.

**Table 6:** Average Euclidean distance between IHC afferent ribbons and nearest efferent terminals in gerbils, naked mole rats, and Damaraland mole rats^a^

Species	Age	Distance (μm)
Gerbil	P9	1.61 ± 0.02 (*N* = 1,074; 3 individuals)
	P42 (6 weeks)	2.15 ± 0.02 (*N* = 1,166, 3 individuals)
Naked mole rat	P11	1.44 ± 0.04 (*N* = 1,074, 3 individuals)
	P365 (1 year)	3.81 ± 0.08 (*N* = 705, 3 individuals)
Damaraland mole rat	P5	3.28 ± 0.13 (*N* = 374, 1 individual)
	Mature years	3.75 ± 0.13 (*N* = 722, 3 individuals)

a *N* = total number of distances and individuals examined.

## 4 DISCUSSION

### 4.1 Overview

In this study, we used immunofluorescence and quantitative image analysis to examine cochlear innervation in mature and developing naked and Damaraland mole rats compared to mice and gerbils. This work was motivated by previous findings that indicate relatively poor hearing in naked mole rats compared to other rodents (Heffner & Heffner, 1993) despite the absence of overt differences in the anatomy of the middle and inner ear in naked mole rats (Mason et al., 2016). In comparison to mice and gerbils, both naked and Damaraland mole rats showed alterations in afferent and efferent innervation as well as their patterns of refinement during development. Differences in cochlear innervation in naked and Damaraland mole rats are likely a consequence of their unusual life histories and offer important comparative and biomedical insights into the mechanisms that underly normal auditory function as well as hearing loss.

### 4.2 Afferent innervation in mature naked and Damaraland mole rats

Examination of afferent innervation in organs of Corti isolated from mature naked and Damaraland mole rats revealed both similarities and differences in comparison to mice and gerbils (Figure 1). Like mice and gerbils, both naked and Damaraland mole rats had segregated inner and OHCs and both types of hair cells contained presynaptic afferent ribbons. Our calculations of the average number of afferent ribbons per IHC in mice and gerbils closely match previously published values (Meyer et al., 2009). Moreover, this work is the first to quantify the average number of afferent ribbons per OHC across these four species. In comparison to mice and gerbils, the average number of afferent ribbons per IHC was reduced in naked and Damaraland mole rats whereas the average number of afferent ribbons per OHC was greater. The ratio of afferent ribbons per IHC compared to OHC is approximately 10:1 in mice and gerbils, 2:1 in naked mole rats, and 1:1 in Damaraland mole rats. Moreover, in contrast to both mice and gerbils, naked mole rats showed no tonotopic variation in the IHC afferent ribbon density (Figure 2).

These afferent ribbons are the presynaptic components of synapses formed with the auditory neurons. Based on extensive research, it is widely accepted that, in mammals, the type I auditory neurons are more abundant and contact single IHCs, whereas the much less abundant type II auditory neurons contact multiple OHCs (Spoendlin, 1972). Thus, the altered ratio of inner and OHC afferent innervation in naked and Damaraland mole rats could reflect a greater proportion of type II auditory neurons and perhaps also a greater number of contacts between the type II auditory neurons and OHCs compared to other mammals. Future work examining the relative abundance of types I and II auditory neurons and their contacts with the hair cells in naked and Damaraland mole rats would provide insight into the generalizability of the pattern of types I and II afferent innervation across mammals.

In addition to quantifying the number of afferent ribbons per hair cell, we also examined differences in the distributions and sizes (volumes) of afferent ribbons between the pillar and modiolar sides of the IHCs. When comparing the relative numbers of modiolar versus pillar afferent ribbons, we observed equivalent numbers of modiolar and pillar synapses in gerbils as well as naked and Damaraland mole rats. In contrast, we observed more modiolar than pillar synapses (56% compared to 44%) in mice, similar to previous reports (Liberman et al., 2011). However, these differences are minor and similar to the differences reported in different studies in the same species. For example, a previous study investigating gerbils reported 57% modiolar ribbons (Zhang et al., 2018) compared to our observation of 50%. Thus, these differences could easily result from subtle differences in the methods of defining the spatial coordinates of ribbons relative to the hair cells, and, therefore, further significance to these differences is not attributed.

When comparing the mean normalized volumes of modiolar versus pillar afferent ribbons, we observed statistically significant differences in mice, gerbils, and naked but not Damaraland mole rats. A difference in size in the same direction, relatively larger modiolar afferent ribbons, has been reported previously in cats (Merchan‐Perez & Liberman, 1996), mice (Liberman et al., 2011), and gerbils (Zhang et al., 2018). Furthermore, in cats, the type I afferent fibers with high thresholds and low spontaneous rates tended to associate with relatively larger ribbons on the modiolar side of the IHC whereas afferent fibers with low thresholds and high spontaneous rates tended to associate with relatively smaller ribbons on the pillar side of the IHC (Merchan‐Perez & Liberman, 1996). The presence of the same spatial gradient in ribbon volumes in naked mole rats suggests that this species may also display a similar distribution of type I afferent subtypes. Future work characterizing auditory responses, and especially the responses of single auditory nerve fibers, in naked and Damaraland mole rats would be necessary to definitively correlate the distribution of IHC ribbon volumes to afferent fiber physiology.

The significance of the increased afferent innervation of OHCs in naked and Damaraland mole rats compared to mice and gerbils is not clear because the function of afferent innervation of the OHCs is poorly understood. Recent *in vitro* experiments indicate that OHCs release vesicles infrequently and their postsynaptic effect is relatively small (Weisz, Glowatzki, & Fuchs, 2009), consistent with earlier *in vivo* recordings suggest that the type II auditory neurons respond only to the loudest sounds or not at all (Brown, 1994; Robertson, 1984; Robertson, Sellick, & Patuzzi, 1999). Increased afferent innervation of the OHCs in naked and Damaraland mole rats would suggest that the type II auditory neurons respond more robustly to sound compared to other rodents. Enhanced activation of type II auditory neurons might, therefore, contribute to the detection and transmission of sound stimuli centrally. The type II, like the type I, central axons project to the cochlear nucleus, where they bifurcate into ascending and descending branches (Brown, Berglund, Kiang, & Ryugo, 1988). Alternatively, or in addition, enhanced activation of type II auditory neurons might contribute peripherally to modulate the local neuronal circuitry of the OHCs, for example, via reciprocal synapses (Thiers, Nadol, & Liberman, 2008) that may play a role in regulating OHC electromotility. Previous observations of poor cochlear tuning in a related mole rat *F. anselli* (Kössl et al., 1996), suggest that the increased OHC afferent innervation (observed in this work) in naked and Damaraland mole rats would not contribute to enhanced cochlear tuning but might even serve to broaden it. Future work examining the structural and functional differences in OHC afferent innervation between naked and Damaraland mole rats and other mammals would help clarify the as yet undetermined role of OHC afferent innervation by type II auditory neurons.

### 4.3 BK channel expression in naked mole rats

Because BK channels in IHCs show variations in their tonotopic distribution along the length of the cochlea and are also associated with the functional maturation of the cochlea, we investigated their expression and tonotopic distribution in naked mole rats (Figure 2). (Additional Damaraland mole rat samples were not available for tonotopic investigation.) Although BK channel immunoreactivity was present in IHCs from naked mole rats, the number and intensity of BK channel immunopuncta per IHC was reduced compared to both mice and gerbils. Furthermore, BK channel expression in IHCs varied tonotopically in both mice and gerbils, peaking in expression density at their respective most sensitive frequencies in the audiogram, 16 kHz in mice and 2 kHz in gerbils and then falling off sharply at higher frequencies. BK channel expression in IHCs was particularly enriched up to 2 kHz in gerbils. In contrast, BK channel expression in IHCs showed no tonotopic variation across apical, middle, and basal turns in naked mole rats. Correspondingly, audiograms of naked mole rats show no particular sensitivity across their range of audible frequencies (Heffner & Heffner, 1993). These findings suggest that BK channel density in the IHCs may contribute to differential sensitivity across frequencies in the cochlea in ways that require further investigation. Finally, BK channels are also expressed in the OHCs of mice (Engel et al., 2006; Maison, Pyott, Meredith, & Liberman, 2013; Rohmann et al., 2015; Wersinger et al., 2010). In this study, BK channels were not detected in the OHCs of naked mole rats. However, we have previously reported that BK channel immunoreactivity in OHCs is sensitive to the duration of fixation (Wersinger et al., 2010). Therefore, the absence of BK channel expression in OHCs in naked mole rats should be investigated more carefully in future experiments.

### 4.4 Efferent innervation in mature naked and Damaraland mole rats

In addition to examining patterns of afferent innervation, we also examined lateral and medial efferent innervation in organs of Corti isolated from mature naked and Damaraland mole rats (Figure 3). All species showed lateral efferent innervation in the region below the IHCs (Figure 3 upper panels). Although the diffuse pattern of innervation made quantification of efferent density difficult, we did detect variations in lateral efferent density among the four species investigated and significantly reduced lateral efferent innervation in naked mole rats compared to both Damaraland mole rats and gerbils. The functional consequences of this reduced lateral efferent innervation are difficult to predict because the role of the lateral efferent system is not well understood. Lateral efferent innervation has been proposed to facilitate binaural hearing (Darrow, Maison, & Liberman, 2006; Irving, Moore, Liberman, & Sumner, 2011), modulate afferent excitability (Groff & Liberman, 2003), and protect the cochlea from noise‐induced injury (Darrow, Maison, & Liberman, 2007). Finally, circuits within the lateral efferent system employ various neurotransmitters and show differences in their patterns of origination within the brain and termination within the cochlea (reviewed in Reijntjes & Pyott, 2016). Therefore, future work should additionally examine possible differences in the neurotransmitters and patterns of innervation employed by the lateral efferent system in naked and Damaraland mole rats. Differences in these patterns may permit a comparative approach to identify the functional contributions of neurotransmitter‐specific lateral efferent circuits.

In addition to lateral efferent innervation, all species examined showed medial efferent innervation of the OHCs (Figure 3 lower panels). Although we did observe medial efferent terminals in naked and Damaraland mole rats, these terminals were smaller and less distinct than the medial efferent terminals observed in mice and gerbils. The diffuse pattern of innervation made quantification of the efferent density difficult; however, we did detect significantly reduced medial efferent innervation density in naked mole rats compared to all other species, including Damaraland mole rats. In comparison to lateral efferent innervation, the role of medial efferent innervation is much better understood. Specifically, medial efferent innervation provides inhibitory input to the OHCs and, thereby, regulates the cochlear amplifier. In this way, medial efferent innervation is believed to improve signal transduction, enhance signal detection, and protect the cochlea from noise‐induced injury (reviewed in both Guinan, 2018; Lopez‐Poveda, 2018). Thus, our findings of reduced medial efferent innervation density are consistent with previous reports of reduced cochlear tuning in a related mole rat species, *F. anselli* (Kössl et al., 1996). The observation of medial efferent innervation in the two mole rats examined in this study, however, contrasts previous ultrastructural observations in another mole rat species, *Spalax ehrenbergi*, in which no efferent innervation of the OHCs was observed (Raphael, Lenoir, Wroblewski, & Pujol, 1991). Thus, the patterns of medial efferent innervation appear to show considerable variation among subterranean rodents.

Finally, although the organization of the hair cells was not specifically investigated, the distribution of IHC afferent ribbons (Figure 1a upper panels) and lateral efferent terminals (Figure 3a upper panels) in naked and Damaraland mole rats indicate one row of IHC, like mice and gerbils. The clustered organization of OHC afferent ribbons (Figure 1a lower panels) in naked mole rats indicates that, at least in middle turns, there are three rows of OHCs, like mice and gerbils, although the medial efferent terminals (Figure 3a lower panels) are much less organized into distinct rows. In contrast, both the OHC afferent ribbons and efferent terminals in Damaraland mole rats were much less organized, at least in middle turns, and suggest three to four rows of OHCs. Precise organization of the OHCs and their hair bundles is necessary for cochlear amplification (LeMasurier & Gillespie, 2005). Therefore, future work in naked and Damaraland mole rats should specifically examine the organization of the OHCs and their bundles as part of physiological assessment of their function.

### 4.5 Development of afferent and efferent innervation in the naked and Damaraland mole rats

In mammals, afferent and efferent innervation of the cochlea undergo extensive modification during development. We examined changes in innervation at ages known or suspected to be before and after the onset of hearing. In this way, we sought to identify comparative differences in the patterns of maturation that might result in functional differences in mature naked and Damaraland mole rats and to obtain possible correlates for the onset of hearing in these animals to guide future experiments. We examined two specific indicators of postnatal maturation: pruning of IHC and OHC afferent ribbons (Figure 4) and loss of efferent innervation of the IHCs (Figure 5).

Pruning of afferent synapses in the developing organ of Corti is analogous to the pruning of supernumerary synapses that occurs elsewhere as a classic feature of nervous system development. This pruning is qualitatively and quantitatively observable in mice and gerbils (Figure 4). In contrast, naked and Damaraland mole rats undergo reduced postnatal pruning of IHC and OHC afferent ribbons. In fact, IHC afferent ribbons are not significantly reduced in Damaraland mole rats. Although we cannot exclude the possibility that pruning occurs prenatally, these findings indicate only subtle postnatal changes in IHC afferent innervation in naked and Damaraland mole rats. Nonetheless, the volume of individual IHC afferent ribbons were significantly increased postnatally in all four species examined. Developmental changes in ribbon numbers and volumes are consistent with previous observations in developing hair cells (Sobkowicz et al., 1982; Wong et al., 2014), which have led to the suggestion that smaller and more numerous ribbons in immature cells merge to form larger and less numerous ribbons in mature cells. Our findings in Damaraland mole rats, in which there is a developmental increase in ribbon size without a change in total ribbon number, suggest that the developmental increase in IHC afferent ribbon volumes occurs independently from the developmental pruning of IHC afferent ribbons.

We also examined postnatal pruning of OHC afferent ribbons. As in mice and gerbils, OHC afferent ribbons were significantly reduced in naked mole rats. In contrast, Damaraland mole rats showed similar numbers of OHC afferent ribbons at both the immature and mature ages. In our data, OHC afferent ribbon pruning is much more prolonged in naked mole rats but nevertheless certainly complete between P28 and 6 months of age. This prolonged period of maturation compared to mice and gerbils may reflect the prolonged retention of an immature pattern of afferent innervation into adulthood, paralleling observations in the naked mole rat brain (Penz et al., 2015).

As a second indicator of postnatal maturation, we examined retraction of efferent terminals from the IHCs (Figure 5). This retraction results from the loss of medial efferent terminals directly contacting the IHCs and consolidation of lateral efferent terminals beneath the IHCs. We quantified retraction as a developmental increase in the distance between the IHC afferent ribbons (located at the base of the IHCs) and their nearest neighboring efferent terminals. As validation of this methodological approach, we observed significantly greater distances between the IHC afferent ribbons and nearest neighboring efferent terminals in mature compared to immature gerbil. The same was also observed in mature compared to immature naked mole rats. In contrast, there was no evidence of retraction in Damaraland mole rats. In fact, the mean distances suggest that, in Damaraland mole rats, efferent innervation is already consolidated early in postnatal development or that there is simply no postnatal axosomatic efferent innervation of the IHCs. Again, because only one immature (P5) Damaraland mole rat specimen was available, these conclusions are made cautiously. Transient efferent innervation of the IHCs likely serves to shape activity‐dependent maturation of the auditory system (Clause et al., 2014). Future work would be necessary to determine how changes in the time course or pattern of this efferent innervation contribute to maturation of the auditory system, and specifically maturation of afferent innervation, in naked and Damaraland mole rats.

## 5 CONCLUSION

For comparative biologists, this work establishes naked and Damaraland mole rats as species worthy of future investigation to examine cochlear mechanisms that enhance frequency sensitivity and sound localization, maturation of the auditory system, and also evolutionary adaptations occurring in response to subterranean environments. For biomedical researchers, naked mole rats, which are extremely long‐lived and highly resistant to various diseases of aging, may also prove particularly valuable to identify mechanisms that prevent or reduce age‐related hearing loss, which involves loss of cochlear function and is one of the most prevalent chronic conditions of aging in humans (Gates & Mills, 2005).
